# Maternal epigenetic responsibility: what can we learn from the pandemic?

**DOI:** 10.1007/s11019-022-10094-z

**Published:** 2022-06-15

**Authors:** Ilke Turkmendag, Ying-Qi Liaw

**Affiliations:** 1 Newcastle Law School, 21 - 24 Windsor Terrace Law School, Newcastle University, Newcastle upon Tyne, NE2 4HQ UK; 2 Warwick Medical School, University of Warwick, Coventry, CV4 7HL UK

**Keywords:** Maternal effects, Epigenetics, Responsibility, Pregnant women

## Abstract

This paper examines the construction of maternal responsibility in transgenerational epigenetics and its implications for pregnant women. Transgenerational epigenetics is suggesting a link between maternal behaviour and lifestyle during pregnancy and the subsequent well-being of their children. For example, poor prenatal diet and exposure to maternal distress during pregnancy are linked to epigenetic changes, which may cause health problems in the offspring. In this field, the uterus is seen as a micro-environment in which new generations can take shape. Because epigenetics concerns how gene expression is influenced by the social realm, including a range of environmental conditions such as stress, diet, smoking, exercise, exposure to chemicals, pollution, and environmental hazards, the research findings in this area have direct policy relevance. For policy makers, rather than controlling this complex range of determinants of health, isolating and targeting maternal body and responsibilising mothers for the control of this micro-environment might seem feasible. Yet, examining the maternal body in isolation as a powerful environment to shape the health of next generations not only responsibilises women for the environment that they cannot control but also makes them a target for intrusive and potentially exploitative biomedical interventions. Even though ‘social factors’ are increasingly considered in epigenetics writing, the phrase is usually taken as self-explanatory without much elaboration. Drawing on the Covid-19 pandemic, this paper moves the current debate forward by providing consolidated examples of how individuals, including pregnant women, have little control over their environment and lifestyle. As evidenced by the pandemic’s disproportionate effects on people with low socioeconomic or poor health status, some pregnant women bore considerable physical and psychological stress which combined with other stress factors such as domestic violence.

## Introduction

In June 2021, the World Health Organisation (WHO) published the first draft of its action plan (2022–2030) to strengthen the implementation of the ‘Global Strategy to Reduce the Harmful Use of Alcohol’. The report included an action plan to prevent consumption of alcohol for vulnerable groups. A statement in the report was met with immediate backlash on social media (on Twitter in particular by a number of feminist academics) and news coverage, because of the definition of this vulnerable group which included ‘women of childbearing age’.^1^ Specifically, the report identified “one of the most dramatic manifestations of harm” of the use of alcohol to others to be “prenatal alcohol exposure and the development of foetal alcohol spectrum disorders”.^2^ The report later said,

appropriate attention should be given to prevention of the initiation of drinking among children and adolescents, prevention of drinking among pregnant women and women of childbearing age, and protection of people from pressures to drink, especially in societies with high levels of alcohol consumption where heavy drinkers are encouraged to drink even more.^3^

Hence, the report suggests that women of reproductive age, which is between 15 and 49 years as defined by the WHO,^4^ regardless of their reproductive plans, should prevent drinking alcohol. There was however no reference to the paternal effect of alcohol consumption in the report, even though it is known that alcohol intake can alter sperm count, size, shape and motility^5^ and that paternal alcohol exposure may increase the risks of birth abnormalities in offspring.^6^ This kind of risk messaging that targets women of reproductive age and pregnant women is not new. For example, maternal impression—the notion that the thoughts, behaviours, emotions and experiences of a pregnant woman could ‘imprint’ on her offspring—“has stubbornly crossed different cultural and medical frameworks from ancient and early modern biology into the early twentieth century”.^7^ But such notions are now backed by findings from epigenetics. Epigenetics explains how environment, lifestyle and behavioural experiences influence the expression of our genes, and how these changes are transmitted to subsequent generations. Transgenerational epigenetics, which explores the mechanisms that transmit the epigenetic information through the germline is one of the most intriguing area of the field; and in recent years, there has been an increase in the research into linking early development to epigenetic events before and during pregnancy.^8^ The intake of alcohol by women during their ‘childbearing age’ and its influence on epigenetic changes is just one example of such research.^9^

Recent research in epigenetics raises complicated questions about maternal responsibility for the health of their offspring and the next generations. Emerging epigenetic research findings on the impact of maternal behaviour on their offspring’s health and wellbeing are translated into prescriptions and proscriptions about new parental responsibilities targeting human mothers, making the pregnant body “the key environmental target for intervention”^10^ and pregnant women responsible for the control of their uterus—the ‘micro environment’ where baby develops. This control deems so important that some scientists argue that rather than waiting for targeted epigenetic treatments which may emerge in the future, a ‘lifestyle epigenetics’ including diet, stress management, and exercise, should be implemented now to improve the health of the population in general, and particularly high-risk populations^11^ (which would include pregnant women). The WHO’s warning for women of reproductive age is an example of the risk messaging targeting women’s reproductive bodies. Even if WHO are much aware of the importance of the social context on individual behaviour (e.g. in the report, it acknowledges the ‘prevailing social norms that support drinking behaviour’),^12^ that kind of statement/risk messaging highlighted earlier may deliver the wrong impression on individuals’ accountability and put women under pressure, thus bringing an *unintended* consequence of stigmatising and responsibilising ‘women of childbearing age’. Furthermore, even if the social context is acknowledged, it does not necessarily mean that such risk messaging by WHO in preventing women of childbearing age not to drink is a laudable public health strategy, just as illustrated by the reaction in the backlash to that statement.

In this paper, we first explore the link between epigenetic risk messaging and the construction of ‘maternal epigenetic responsibility’ in Parts 2 and 3. Particularly, we explain how the maternal body is considered a closed system, a micro-environment for foetal development and often, women are responsibilised for controlling this to optimise the condition of the environment where the baby is developing. We also highlight the critics of this approach that emphasise that the maternal body is an embedded body, and it is situated in a social and physical environment that is difficult to control, especially for those who do not have the means to do so. In Part 4, we contribute to the existing debate by bringing evidence to support the critics by drawing on studies that explored the Covid-19 pandemic’s unprecedented and unpredictable effects on expectant mothers. We observe that during the pandemic, women’s bodies continue to be deemed a better realm to be investigated than other influences (e.g. fathers) on the baby’s development. We also argue that the pandemic at the same time serves as a good example of how little control individuals have over their environment, social circumstances, as most of us were locked down in our own micro-environment.^13^ Therefore, epigenetic responsibility should not be individualised. Instead of attributing moral responsibility to mothers for public health, we should consider that individuals have little control over their environment, social circumstances, and the wider socio-economic inequalities that affects them.

## Epigenetics risk messaging and the maternal body

As indicated earlier, the findings in epigenetics suggesting a link between maternal behaviour and experiences during pregnancy and after birth, and the subsequent well-being of their children and the future generations. Apart from alcohol intake before conception and during pregnancy, other examples including poor prenatal diet,^14^ prenatal exposure to domestic violence or maternal distress,^15^ Caesarean delivery,^16^ are all linked to epigenetic changes which may cause health problems in the offspring.

The research into epigenetic mechanisms might provide important insights into foetal development, but there is a problem that was identified by the social scientists 17 In epigenetic studies examining maternal effects, a pregnant woman’s body is treated as a vessel in which future generations can be shaped and hence, “women’s bodies become de facto sites for research and intervention in relation to the health of her offspring”.^18^ Kenney and Müller note, “simplified and remarkably stereotypical notions of maternal agency and responsibility often travel between contexts without much scrutiny and are, in the process, reinforced and solidified rather than critically questioned and opened up for novel interpretation”.^19^ Furthermore, current epigenetics research “work to *illustrate* rather than *interrogate* existing stereotypes about maternal agency and responsibility (emphasis added).”^20^ There is also a critic of the accuracy and representativeness of these studies. The multiple translation of research across different species (e.g. rat, mice and humans) and different disciplines is problematic.^21^ The understanding of environmental targets for prenatal interventions in humans draw on animal models such as rats in a strictly controlled environment, while other aspects of environment such as housing, toxic exposure, and stress are not considered.^22^

Although many scientific findings in epigenetics are too preliminary to provide a solid evidence base for recommendations to change daily living, they are turned into sensationalist headlines by the media addressing expectant mothers and are used to support various public health interventions targeting pregnant women^23^ as well as being integrated into risk-reduction protocols in clinical practice, particularly in reproductive health care.^24^ In such settings, pregnant women or mothers seem to bear the responsibility for their future children’s health. For instance, consider the headline such as ‘*What a woman eats BEFORE she becomes pregnant affects her child's health for life*’ which suggests a burden of responsibility for women to take care of their diets even before conception for the sake of their future offspring^25^; in ‘*Mother’s stress harms foetus, research shows*’, it is highlighted the need for pregnant women “to lead a healthy, balanced lifestyle to avoid general stress”^26^; also, in ‘*Stress in pregnancy may influence baby brain development*’, it is urged that more support to be given to pregnant women as it could be “an effective way of promoting healthy brain development in their babies”.^27^ Although mothers are often treated as a micro ecosystem for their foetuses,^28^ this kind of epigenetic risk messaging creates a new form of responsibility for women of ‘childbearing age’. This is maternal epigenetic responsibility: a responsibility for women at reproductive age to control, protect and nourish their reproductive health to create the best micro-environment for their potential offspring and the next generations.

## Maternal epigenetic responsibility

Since epigenetics encompasses adoptability, flexibility and plasticity in that it concerns how gene expression is influenced by a range of environmental conditions such as stress, exposure to chemicals, pollution and environmental hazards, some commentators see epigenetics as a paradigm shift in interaction of nature and nurture, offering a more holistic understanding: it is viewed as a “biosocial approach” to health, considering the biological and social as mutually constituting processes of human development over the life course.^29^ This makes epigenetics an attractive tool that reflects a popular health discourse which focuses on control and self-improvement. For policy makers, rather than controlling a complex range of determinants of health, isolating and targeting maternal body as a micro-environment and responsibilising mothers for the control of this realm might seem feasible. This raises the question of maternal epigenetic responsibility. Hedlund observes that the progress made in the field of epigenetics might “contribute to an emphasis on individuals’ responsibility for their own health and the health of future descendants” and lead to “the risk of stigmatisation of those who do not take responsibility for their own health”.^30^ The claims associated with new knowledge of epigenetics therefore create novel ethical challenges regarding the moral responsibility of the individual,^31^ the relationship between the state and citizens^32^ as well as potential surveillance and discrimination of risk groups, including women at reproductive age.^33^

Hedlund argues that the most fundamental issue for the societal governance of epigenetics is that of responsibility.^34^ Yet, the implications of epigenetics for collective responsibility and individual responsibility are not always clear. It is noted that approaches to monitor and dismiss or penalise certain behaviour of pregnant women may imply an over-individualistic approach to responsibility; responsibility towards children implies “at least an enabling society” and striking a balance between individual and collective responsibility remains unresolved.^35^ The findings from epigenetics further complicate the issue of responsibility. As Hedlund rightly questions, given the social environment cannot be controlled by individuals, should collective actors be responsible for protecting the individuals against environmental hazards?^36^ How about the “micro-environment” a pregnant woman creates for her foetus?^37^ Considering the implications by epigenetics of early intrauterine life, should she be responsible for harmful epigenetic effects on her child before she even knows she is pregnant?^38^ The transgenerational aspect of epigenetics may also add up to the existing complications in that it may increase responsibilities to a demanding level. Hens nicely exemplifies this complexity:

what a pregnant woman is exposed to may influence the germ cells of the foetus she is carrying, and thus affect the health of the children of that foetus, her grandchildren. Living in polluted areas may induce epigenetic changes and affect offspring, even after families have moved to non-polluted regions. (…) The responsibility could be assigned to either the person herself or those who cause the pollution, or could be conceived as a shared responsibility. We may have a collective responsibility towards all future generations, but how we must exercise that responsibility and how far this individual responsibility extends is unresolved.^39^

Given that epigenetic knowledge may further pressure expectant mothers who already are bearing a “disproportionate responsibility” due to the current social expectations on maternal responsibilities towards their future offspring,^40^ it is essential to report and use the epigenetics-related information sensibly. In 2014, a group of social scientists published a commentary in Nature urging scientists, educators and reporters to anticipate how the research in the burgeoning field of epigenetic studies of the developmental origins of health and disease (DOHaD) linking certain forms of maternal behaviour with the health of their offspring is likely to be interpreted in popular discussions.^41^ Speculative, oversimplified, and exaggerated research findings may form the basis for increased surveillance and regulation of pregnancy, consequently extending biomedical manipulation and social control of the female reproductive body.^42^ This is very similar to the way in which neuroscience has been simplified and translated into a set of evidence-based, targeted programmes on parenting.^43^ Previous social science research on neuroscience has illustrated the dangers of naïvely using new scientific evidence as the basis for policy, which include the medicalisation of policy debate, suppressing necessary moral discourse and pushing practice towards systematised and targeted interventions whereas intervention in social context such as providing family and community support has largely ignored.^44^ Thus, in the epigenetics context, one should be cautious in using the knowledge for attributing individual moral responsibility on mothers by having targeted interventions towards them. Rather, we should put more attention on the social structures that significantly affect one’s nutrition, lifestyle, physical and mental health issues.

As we discuss in the following, the global pandemic of Covid-19 shows us how little control we have over our environment and circumstances including management of stress, exposure to domestic violence, and access to medical care on demand. Accordingly, we argue that as the emerging discourses of epigenetics may increase social surveillance of mothers and the regulation of pregnancy,^45^ ‘epigenetic risk messaging’^46^ is ultimately more oppressive than it is empowering, especially for women who have little or no control of their environment.

## Pregnancy in covid-19 pandemic

As indicated in the previous section, much attention has been drawn to maternal behaviour and lifestyle before, during and after pregnancy in transgenerational epigenetic studies, reflecting an apparent assumption of maternal responsibility in the offspring’s subsequent well-being. While there seems to be a wider acknowledgement of social contexts by several authors in epigenetics,^47^*how this social context comes into play is not always clearly explained or elaborated*. This paper fills this gap by providing a case study of the complex causality relations using Covid-19. In particular, the current paper contributes to the existing research in two ways: (i) by illustrating that mothers continue to be under the spotlight in the pandemic; and ii) by elaborating on the social factors with consolidated examples using the pandemic. A more detailed explanation of how social factors operate will provide further insights for the policymakers to better understand the possible root causes of the issues at hand and more carefully draft a risk message affecting mothers or pregnant women without, intentionally or unintentionally, responsibilising them. We hope that this research will be a strengthened evidence on why there should be more critical and better reporting on transgenerational epigenetics findings. We suggest that although the Covid-19 pandemic may have generated information for invaluable research to better apprehend the causes and development of diseases such as the neurodevelopmental disorders, the prevailing focus on maternal effects at the time of pandemic also implies that women’s bodies continue to attract researchers’ interest and be captured as a biomedical tool for research insights, thus in a way reinforcing the view of women bodies as micro-environments. We highlight that as evidenced by the Covid-19 pandemic’s disproportionate effects on people with low socioeconomic or poor health status, individuals including pregnant women at times have little control over their environment and lifestyle. Can we really hold pregnant women responsible for not creating the best possible environment for their babies when they are trapped in circumstances that are beyond their control?

### The continuation of assumption of maternal responsibility in Covid-19-related research

In the field of epigenetics, there is an imbalance between the number of studies that explore maternal and paternal effects. Richardson argues that scientists defend this disparity by stressing the importance of the maternal phenotype which has a greater capacity to shape the offspring’s phenotype. Yet, there are wider reasons behind this which “originates in long-standing Western cultural and ideological convictions” including a belief in the primary liability of the mother for infant care and development and a resistance to the notion of paternal responsibility for the embryo development.^48^ Accordingly, Sharp, Lawlor and Richardson have urged the researchers to remain critical over the deeply held assumption that there is a great causal relation between maternal experience or exposure around the time of pregnancy with the health outcomes of future children.^49^ Still, a similar trend focusing on maternal effects (instead of paternal factors) has become discernible in Covid-19-related research. An unfiltered search on PubMed and Scopus database on terms (“Covid-19”) AND (“pregnant women” OR “mother” OR “maternal”) shows an outcome of 4923 and 23,508 results respectively compared to (“Covid-19”) AND ("father" OR “paternal”) which reveals only 135 and 2448 results respectively (both search results as of February 4, 2022). In particular, Covid-19 pandemic is seen as a unique opportunity for researchers to study (or perhaps more accurately, verify) the relationship between prenatal maternal infection, maternal behaviour, experiences or emotions (e.g. maternal stress) and the development of resulting offspring. For instance, in a study by Ayesa-Arriola and others, the authors write:

Under this scenario, approximately 100 million pregnant women would be at potential risk of acquiring the SARS-CoV-2 infection, and therefore, *there is growing concern about a dramatic increase of neurodevelopmental problems in the offspring of mothers infected during pregnancy in the coming years*, similar to that observed after the 1918 influenza pandemic. From a wider viewpoint, this concurrence could provide *a unique opportunity for advancing and refining the hypothesis of how prenatal exposure to infection might jeopardize normal brain development*, increasing the likelihood of later neuropsychiatric disorders.^50^ (Emphasis added)

Apart from the consequences of prenatal maternal infection, the pandemic also presents a window of opportunity for researchers to examine “a unique form of prenatal stress in real time”.^51^ The *effects* of maternal stress during pregnancy and especially during this pandemic are consistently highlighted. For instance, Saxbe and Morris, while highlighting an upsurge in depression for women pregnant during this pandemic due to limited social connection, constrained prenatal health care, income and employment, suggest that “although infants born in 2020 may not remember the pandemic first hand, *its effects may shape their early lives* in ways that we are just beginning to measure” 52 (our emphasis). In another study conducted by Nabi and colleagues, the authors also observe that Covid-19 induced psychosocial stressors (such as isolation, restricted social activities, troubled sleeping, lockdown) during gestation and point out that maternal psychosocial stress can affect foetal brain development and maturation. The authors therefore call for prompt psychological interventions “to resist the development of maternal mental aberrations as well as reducing the risks of foetal and child somatic and mental disorders”.^53^

The flip side of what seems to be an empowerment for pregnant women (in the sense of having control and support in coping with stressful event) is an assumption of responsibility that such studies may have, unintentionally, fuelled towards pregnant women.^54^ Mothers’ frustration and their concerns for their babies can be sensed in a qualitative study conducted by Mortazavi and Ghardashi (albeit their findings are not directly related to epigenetic effects but the lived experiences of pregnant women during the pandemic): e.g. two women were reported to have said,

I was in my 5th month in March when I saw a clip of infected women who had successfully given birth and were being treated. I was constantly stressed by not know what would happen to my unborn baby if I were to get sick. Would I have to abort? Would my child become sick too?^55^;

What can we do with so stress these days? With these stresses, how we can be healthy women and give birth to a healthy child?^56^

In these accounts, women express both anxiety about the unknown effects of Covid-19 on their future baby’s health and uncertainty about their moral responsibility as mothers. Should they terminate the pregnancy if they get sick? Would the stress caused by the pandemic affect their baby’s health? While there is already research indicating potential transgenerational epigenetic traumatic stress due to Covid-19,^57^ it remains unanswered how these women might feel about the risk messaging from the epigenetics studies in relation to the pandemic. Would mothers feel guilty or be blamed for choosing to conceive during the pandemic with heightened stress and with a risk of getting infected by the virus that could, as the epigenetics studies suggest, have long-term effects to future offspring? This is a sensible and important question to be asked considering that in other context such as the awareness of epigenetic changes up to four generations due to exposures to smoke has created “a heavy responsibility on the pregnant woman not to smoke”.^58^ However, as we discuss below, this is not a straightforward issue.

### Covid-19 pandemic: a magnified illustration of the complex causality relations

Hedlund questions if it is reasonable to hold individuals responsible for repercussions of their way of life “given the social, economic and political structures in which they make their lifestyle choices”.^59^ The Covid-19 pandemic well-illustrates the complexity in establishing the causality relations described by Hedlund, which, as we argue, casts doubt on the actual chances for pregnant women or mothers to make free and voluntary choice over their lifestyle. According to Hedlund, “social, economic, political and other material and ideational structural conditions would also affect epigenetic processes, directly by contributing to physical and mental stress and wellbeing, indirectly by constraining and enabling individual factors”.^60^ In considering the impacts of psychosocial stress on epigenetic profiles and health, it is suggested that socially disadvantaged groups are likely to face higher risk of exposure to psychosocial stressors and hence suffer from adverse disease outcomes.^61^ As we shall show, the Covid-19 pandemic is a solid reminder of factors outside our (including pregnant women’s) control as we go on our daily life.

#### Stress, anxiety and domestic violence

As indicated above, the Covid-19 pandemic has heightened mental health issues to individuals including pregnant women. Studies show that pregnant women were highly anxious about being infected in the time of pregnancy and childbirth.^62^ However, even when women have taken precaution to reduce the risk of infection themselves, there is still a fear of infection which derives from the behaviour or daily activities of their partner. Consider the following:

I take care of myself but my husband go(es) out. What can I do if he gets the virus and transmits it to me?^63^;

I’m more concerned for my husband and my parents than myself. I was in home-quarantine, but my husband had to leave every day for work. His job has him be in contact with many people.^64^;

My husband would wear a mask whenever he left the house, but he didn’t care as much as I did, and that would add to my worries.^65^

This shows that there are multiple sources that may generate stress and anxiety for the women (be it the disaster itself or due to the behaviours of their partner). As such, messages urging women to live a less stressful life is not a way forward without attending to other factors.

Moreover, social media is also identified as a source of stress and anxiety during the pandemic. Women who followed the pandemic outbreak on social media and television were bombarded with disappointing news that led to more stress and fear.^66^ For instance, a woman said,

We were so scared whether we heard it on social media or on television and talking to friends, you know, everyone was spreading all this hearsay nonsense information…^67^

In fact, it is observed that the “social media panic” spread faster than the virus during the pandemic and this is due to misinformation on the coronavirus that consequently causes confusion and fear.^68^ Hence, it is important to focus on how certain information is *presented* to deliver a message right.^69^ The role of news and social media is relevant to this paper in two aspects. First, one can argue that it is beyond individuals’ (including pregnant women’s) control on how the information are presented to them through news or social media and may (or may not) generate a stress factor for them. This is relatable not just during this pandemic but in life generally especially in this era where there is great advancement in communications technology. Exposure to media coverage of negative and sensational news is seen to have great impact on individuals’ psychological distress.^70^ Second, in relation to epigenetic findings and how they are reported in popular media, current emphasis on parents’ experiences (mostly maternal experiences) may disproportionately influence perception of parents’ (particularly, expectant mothers’) responsibilities for the health of offspring while downsizing the role of wider social contexts and structural issues that often shape the experiences.^71^ We continue to demonstrate the role of these wider considerations in the rest of the discussion using the Covid-19 pandemic.

Some pregnant women bore considerable physical and psychological stress which combined with other stress factors to which they cannot be accountable for, such as domestic violence. The claim of an increased risk of family violence especially against women during the pandemic is well-founded.^72^ Usher and others warn about the mental health implications of family violence (including intimate partner violence, domestic abuse and domestic violence) and describe Covid-19 pandemic as the “perfect storm” for family violence in which the perpetrators of violence can manipulate the situation for coercion and control (e.g. tracking the daily activities/movement of their partners).^73^ For families where the risk of violence is imminent, it is sceptical to reasonably expect pregnant women to handle or reduce stress for the sake of their future offspring when they are trapped in isolation with an abuser.

Nonetheless, it is also important to note that these burdens do not apply equally to all individuals and in reality, not every woman has the capacity to keep stress at bay for their future children, even if they want to. Especially in the context of pregnant women, it is observed that the disruptions caused by the pandemic such as those related to prenatal care, lack of access to technology, lack of safe spaces to conduct telehealth may contribute to substandard birth outcomes and these are differentially affecting the minority communities.^74^ Thus, the Covid-19 pandemic certainly magnifies the “longstanding structural drives of health inequities” including subpar working conditions, increasing economic disparities which all connected with ethnicity, gender, class, education level and other factors during the pandemic.^75^ In other words, with stress and adversity seen in the pandemic, not every individual is equipped with the resources in place to protect themselves against these factors—some “may lack these resources and/or have characteristics that predispose them to greater stress and adversity”.^76^

#### Healthy lifestyle and diet

With the restrictive movement in place because of the Covid-19 pandemic, pregnant women experience a change of daily routines, including daily walking exercises. For instance, some pregnant women were reported saying,

What’s pushing me is that I'm home all the time, my body is still inactive. I couldn't walk or anything, but now I can't, and I have increased pain in my legs due to immobility^77^;

Yeah, it's kind of challenging to stay home, so for example, I was going for a walk, but now I can't, I'm always home.^78^

While Covid-19 has presented barriers to physical activity among pregnant women to which they have little control of, such barriers exist (in many different forms) even without the pandemic. These barriers include the safety of physical exercise in which some women in certain ethnic and cultural groups have raised concerns, cultural norm of lack of exercise habits, lack of affordable and safe places for physical activity, absence of social support from family or friends, insufficient information (or a lack of awareness) and work accountabilities.^79^ Hence, simply encouraging “exercise during pregnancy” so as to “save kids from health problems as adults”^80^ based on epigenetic findings sends a wrong message on the responsibility of pregnant women for it neglects the interpersonal and environmental factors that may hindered have exercising in the first place.

Maternal diet is also heavily affected throughout the pandemic. For instance, Dolin and colleagues observe that food insecurity—that is, restricted or varying availability of nutritionally adequate and safe food^81^—has been aggravated during the pandemic.^82^ In particular, the pregnant women within the minority communities are seen to share the unequal burden of the crisis of food and nutrition insecurity.^83^ Barbosa-Leiker and others suggest that food scarcity, lockdown restrictions coupled with limited social support in “perinatal women of racial and ethnic minority and lower-income status” made it difficult for pregnant women to obtain healthy food during the pandemic.^84^ Hence, there is no doubt that the pandemic does “discriminate” with those of poor socioeconomic status affected the hardest because one’s socioeconomic status influences where one lives, what one eats, what kind of job one has and whether one has access to adequate health insurance and healthcare; one living conditions also determines the sanitation level, the issue of overcrowding and the ability to appropriately practise physical distance.^85^ All of these suggest that the causal relation of maternal effects on the development of future offspring may not be as direct as first observed in the epigenetics studies because maternal health and lifestyle are heavily influenced by the wider societal, political and environmental factors.

## Conclusion

While the discussion above has focused on the Covid-19 pandemic, the point that we would like to convey—examining maternal body as an isolated environment to shape the health of next generations not only risks responsibilising women for the environment that they cannot control but also makes them a target for interventions—can be well-adapted to a more general context. The wider causal factors pointed out earlier are often overlooked in the epigenetic risk messaging to the public. The neglected question is whether or not pregnant women or mothers are *able* to implement behavioural change such as to lead a healthy lifestyle and to avoid general stress as advocated? For instance, is it realistic for women who feel distressed to resort to counselling service if they are not aware that there is such help out there, or that they need to work long hours to sustain financial resources that they do not have time to self-care, or they are living in a house full of other children or even worse, with a violent partner?

In short, targeting and isolating women’s body as a target for intervention overlooks the fact that in reality, the options for them might be quite limited in that they may be stuck in a position beyond their control. Before ascribing any responsibility to them, it is important to factor in the capacity of women to enact changes and this includes considering the position in which they are embedded, including the social structures that lead to health disparities that they are subject to.^86^ A way forward in the epigenetics research agenda should include identifying and tackling with the root causes of maternal behaviour and lifestyle such as stress and poor prenatal diet, in addition to creating support hubs for women who seek help and support to cope with stress or to maintain a balanced and healthy lifestyle. The role of media coverage is also crucial in reporting epigenetic studies. Individuals and expectant parents, especially pregnant women and young mothers, have a great interest in accessing reliable information about maternal health care and early development. Miscommunication of findings in this area might have negative implications on pregnant women’s mental and physical well-being and burden them in situations over which they have little control. Scientists and science publicists have an important role in communicating the research to the press and public. The successful translation of epigenetics knowledge in practice and policy thus requires greater attention and a meaningful interaction between scientists, social scientists, practitioners, policymakers and publics to ensure the benefits of this important field are realised in a responsible manner.

## Footnotes

1 See for instance, Turkmendag, I. Sins of ‘Women of Childbearing Age’. SLSA Blog, University of Birmingham, (2021). Available at: http://slsablog.co.uk/blog/blog-posts/sins-of-women-of-childbearing-age/ (last accessed 22 March 2022); Campoamor, D. The WHO alcohol-pregnancy warning for childbearing women overlooks men, as usual. *Nbc News* (2021) June 21. Available at https://www.nbcnews.com/think/opinion/who-alcohol-pregnancy-warning-childbearing-women-overlooks-men-usual-ncna1271690 (last accessed 02 February 2022); Javed, S. WHO criticised by pregnancy services for suggesting ‘women of child-bearing age should not drink alcohol. *The Independent* (2021) June 17. Available at https://www.independent.co.uk/life-style/women/who-alcohol-women-pregnancy-report-b1867960.html (last accessed 02 February 2022); Davies, J. WHO plan to stop to stop women of child-bearing age from drinking alcohol would be 'completely disproportionate', critics say. *Daily Mail* (2021) July 2. Available at https://www.dailymail.co.uk/health/article-9696423/Experts-fury-wanting-BAN-women-child-bearing-age-drinking-alcohol.html (last accessed 02 February 2022); Taylor, B. WHO action plan on alcohol sparks confusion, allegations of sexism. *CTV News* (2021) June 19. Available at https://www.ctvnews.ca/health/who-action-plan-on-alcohol-sparks-confusion-allegations-of-sexism-1.5477639 (last accessed 02 February 2022); Bourke, L. No booze until menopause: WHO’s alcohol guidance sparks female backlash. *The Sydney Morning Herald* (2021) June 19. Available at https://www.smh.com.au/world/europe/no-booze-until-menopause-who-s-alcohol-guidance-sparks-female-backlash-20210618-p582e4.html (last accessed 02 February 2022).

2 World Health Organization. Global action plan on alcohol: 1st draft. (2021a) June; available at https://cdn.who.int/media/docs/default-source/alcohol/action-plan-on-alcohol_first-draft-final_formatted.pdf?sfvrsn=b690edb0_1&download=true (last accessed 14 July 2021) 2.

3 See note 2, World Health Organization (2021, p. 17).

4 World Health Organization. Maternal, newborn, child and adolescent health and ageing: Data portal. (2021b); available at https://www.who.int/data/maternal-newborn-child-adolescent-ageing/indicator-explorer-new/mca/women-of-reproductive-age-(15-49-years)-population-(thousands) (last accessed 14 July 2021).

5 Jensen, TK, Gottschau, M, Madsen, JO, Andersson, AM, Lassen, TH, Skakkebæk, NE, et al. Habitual alcohol consumption associated with reduced semen quality and changes in reproductive hormones; a cross-sectional study among 1221 young Danish men. *Reproductive medicine* (2014);4(9):e005462.

6 Zhou, Q, Song, L, Chen, J, Wang, Q, Shen, H, Zhang, S and Li, X. Association of preconception paternal alcohol consumption with increased fetal birth defect risk. *JAMA Paediatrics* (2021);175(7):742–743.

7 Meloni, M, and Müller, R. Transgenerational epigenetic inheritance and social responsibility: Perspectives from the social sciences. *Environmental Epigenetics*, (2018);4(2):dvy019, 3.

8 Valdez, N. The redistribution of reproductive responsibility: On the epigenetics of “environment” in prenatal interventions. *Medical Anthropology Quarterly* (2018);32(3):425–442.

9 Jabbar, S, Chastain, L, Gangisetty O, Cabrera MA, Sochacki K and Sarkar D. Preconception alcohol increases offspring vulnerability to stress. *Neuropsychopharmacology* (2016);41(11):2782–2793.

10 See note 8, Valdez (2018, p. 432).

11 Herbert, MR. Everyday epigenetics: From molecular intervention to public health and lifestyle medicine. *North American Journal of Medicine and Science* (2013);6(3):167–170, 169.

12 See note 2, World Health Organization (2021, p. 1).

13 See Prof Robert Dingwall’s comments in Expert reaction to plans for self isolation for over 70 s that may be implemented in the UK in the coming weeks. *Science Media Centre* (2020) March 15. Available at https://www.sciencemediacentre.org/expert-reaction-to-plans-for-self-isolation-for-over-70s-that-may-be-implemented-in-the-uk-in-the-coming-weeks/ (last accessed on 08 February 2022).

14 See for instance, Geraghty, A, Lindsay, K, Alberdi, G, McAuliffe F and Gibney E. Nutrition during pregnancy impacts offspring’s epigenetic status—evidence from human and animal studies. *Nutrition and Metabolic Insights* (2015);8(Suppl 1):41–47.

15 See for instance, Radtke, KM, Ruf, M, Gunter, HM, Dohrmann K, Schauer M, Meyer, A and Elbert, T. Transgenerational impact of intimate partner violence on methylation in the promoter of the glucocorticoid receptor. *Translational Psychiatry* (2011);1(e21):1–6; also, Cao-Lei, L, Laplante, DP and King, S. Prenatal Maternal Stress and Epigenetics: Review of the Human Research. *Current Molecular Biology Reports* (2016);2:16–25.

16 See for instance, Almgren, M, Schlinzig, T, Gomez-Cabrero, D, Gunnar, A, Sundin M, Johansson, S, et al. Cesarean delivery and hematopoietic stem cell epigenetics in the newborn infant: Implications for future health? *American Journal of Obstetrics and Gynecology* (2014); 211(5):502.e1-502.e8.

17 See Lappé, M. The paradox of care in behavioral epigenetics: Constructing early-life adversity in the lab. *BioSocieties* (2018);13(4); 698–714; Kenney, M. and Müller, R. Of rats and women: Narratives of motherhood in environmental epigenetics. *BioSocieties* (2017);12(1):23–46; Richardson, S. Plasticity and programming: feminism and the epigenetic imaginary. *Signs: Journal of Women in Culture and Society* (2017);43(1):29–52; Richardson, S. Maternal bodies in the postgenomic order: Gender and the explanatory landscape of epigenetics. In: Richardson S and Stevens H, eds. *Postgenomics: Perspectives on Biology after the Genome.* Duke University Press (2015);210–231; note 8, Valdez (2018).

18 See note 17, Kenney and Müller (2017, p. 27).

19 See note 17, Kenney and Müller (2017, p. 30).

20 See note 17, Kenney and Müller (2017, p. 23).

21 See note 17, Kenney and Müller (2017).

22 See note 8, Valdez (2018).

23 See note 8, Valdez (2018).

24 Juengst, ET, Fishman, JR, McGowan, ML and Settersten Jr, RA. Serving epigenetics before its time. *Trends in Genetics* (2014);30(10):427–429.

25 Macrae, F. What a woman eats BEFORE she becomes pregnant affects her child's health for life. *Daily Mail* (2014) April 29. Available at https://www.dailymail.co.uk/health/article-2615985/What-woman-eats-BEFORE-pregnant-affects-childs-health-life-Milk-green-veg-protein-effect-genes.html (last accessed 17 July 2021).

26 Ward, L. Mother’s stress harms foetus, research shows. *The Guardian* (2007) May 31. Available at https://www.theguardian.com/science/2007/may/31/childrensservices.medicineandhealth (last accessed 17 July 2021).

27 University of Edinburgh. Stress in pregnancy may influence baby brain development. *ScienceDaily* (2020) November 24. Available at https://www.sciencedaily.com/releases/2020/11/201124111105.htm (last accessed 17 July 2021).

28 For example, in her book, Duden suggested that advances in screening technology and changes in public discourse have construed the mother as an ecosystem, and the fetus as an endangered species, pitting fetus against mother. Duden, B. Disembodying Women: Perspectives on Pregnancy and the Unborn. Cambridge: Harvard University Press (1993). Similarly, Manderson’s work underscores how women are regarded as “foetal containers”, and how this approach reinforces the idea that women are “vehicles of poor intergenerational health” see Manderson, L. Foetal politics and the prevention of chronic disease. Australian Feminist Studies (2016);31:154–171, 154.

29 Mcdade, TW and Harris, KM. The biosocial approach to human development, behavior, and health across the life course. *The Russell Sage Foundation Journal of the Social Sciences* (2018);4(4):2–26.

30 Hedlund, M. Epigenetic responsibility. *Medicine Studies* 2012;3:171–183.

31 Chadwick, RF and O'Connor, A. Epigenetics and personalised medicine. *Personalized Medicine* (2013);10(5):463–471.

32 See note 30, Hedlund 2012; also, [Meloni, M. and Testa, G. Scrutinizing the epigenetics revolution. *BioSocieties* (2014); 9(4):431–456.

33 Rothstein, MA, Cai, Y and Marchant, GE. The ghost in our genes: legal and ethical implications of epigenetics. *Health matrix* (2009);19(1):1–62.

34 See note 30, Hedlund 2012.

35 Hens, K. Neurological diversity and epigenetic influences in utero. An ethical investigation of maternal responsibility towards the future child. In: Hens K, Cutas D, Horstkötter D, eds. *Parental Responsibility in the Context of Neuroscience and Genetics.* Switzerland: Springer International Publishing 2017;105–119, 107.

36 See note 30, Hedlund 2012.

37 See note 30, Hedlund 2012.

38 See note 30, Hedlund 2012.

39 See note 35, Hens (2017, p. 108).

40 Hessler, K, Epigenetic inheritance and the moral responsibilities of mothers. *AMA Journal of Ethics* (2013);15(9):767–770, 767. Available at https://journalofethics.ama-assn.org/article/epigenetic-inheritance-and-moral-responsibilities-mothers/2013-09 (last accessed 16 July 2021).

41 Richardson S, Daniels, CR, Gillman, MW, Golden, J, Kukla R, Kuzawa, C and Rich-Edwards J. Don't blame the mothers. *Nature* (2014);512:131–132.

42 See note 41, Richardson et al. (2014).

43 Wastell, D and White, S. Blinded by neuroscience: Social policy, the family and the infant brain*. Families, Relationships and Societies* (2012);1(3):397–414.

44 See note 43, Wastell and White (2012).

45 See note 41, Richardson et al. (2014).

46 See note 24, Juengst et al. (2014).

47 See for instance, note 24, Juengst et al. (2014, p. 428). The authors argue that "shifting the expectations for prevention to parents unfairly targets the most socially vulnerable within these groups, creates the potential for social blame, and even exacerbates environment, social, and intergenerational injustices".

48 See note 17, Richardson (2015).

49 Sharp, G, Lawlor, D and Richardson, S. It's the mother!: How assumptions about the causal primacy of maternal effects influence research on the developmental origins of health and disease. *Social Science & Medicine* (2018);213:20–27.

50 Ayesa-Arriola, R, López-Díaz, A, Ruiz-Veguilla, M, Leza, JC, Saura, LF and Crespo-Facorro, B. COVID-19 as a unique opportunity to unravel the link between prenatal maternal infection, brain development and neuropsychiatric disorders in offspring. *Revista de Psiquiatría y Salud Mental (Barcelona)*(2021);14(1):1–3.

51 Saxbe, D and Morris, A. Pregnancy during COVID-19 lockdown: How the pandemic has affected new mothers *The Conversation* (2021) May 19. Available at https://theconversation.com/pregnancy-during-covid-19-lockdown-how-the-pandemic-has-affected-new-mothers-159789 (last accessed 16 July 2021).

52 See note 51, Saxbe and Morris (2021).

53 Nabi, G, Siddique, R, Xiaoyan, W, Ullah, R, Nawsherwan, Xue, M and Khan, S. COVID-19 induced psychosocial stressors during gestation: possible maternal and neonatal consequences. *Current Medical Research and Opinion* (2020);36(10):1633–1634.

54 Vears, D and D’Abramo, F. Health, wealth and behavioural change: An exploration of the role of responsibilities in the wake of epigenetics. *Journal of Community Genetics* (2018);9(2):153–167.

55 Mortazavi, F and Ghardashi, F. The lived experiences of pregnant women during COVID-19 pandemic: A descriptive phenomenological study. *BMC Pregnancy and Childbirth* (2021);21:1–10, 4.

56 See note 55, Mortazavi and Ghardashi (2021, p. 5).

57 Tang, H, Tanaka, G and Bursztajn, HJ. Transgenerational transmission of resilience after catastrophic trauma. Psychiatric Times (2021);38(6). Available at https://www.psychiatrictimes.com/view/transgenerational-transmission-of-resilience-after-catastrophic-trauma (last accessed 04 February 2022).

58 See note 36, Hessler (2013).

59 See note 30, Hedlund (2012).

60 See note 30, Hedlund (2012).

61 Thayer, ZM and Kuzawa, CW. Biological memories of past environments: epigenetic pathways to health disparities. *Epigenetics* (2011);6(7):799, 800.

62 See note 55, Mortazavi and Ghardashi (2021); also Sahin, BM and Kabakci, EN, The experiences of pregnant women during the COVID-19 pandemic in Turkey: A qualitative study. *Journal of the Australian College of Midwives* (2020); 34(2):162–169.

63 See note 55, Mortazavi and Ghardashi (2021, p. 5).

64 See note 55, Mortazavi and Ghardashi (2021, p. 5).

65 See note 55, Mortazavi and Ghardashi (2021, p. 5).

66 See note 55, Mortazavi and Ghardashi (2021, p. 8).

67 See note 62, Sahin and Kabakci (2020).

68 Depoux, A, Martin, S, Karafillakis E, Preet, R, Wilder-Smith, A and Larson, H. The pandemic of social media panic travels faster than the Covid-19 outbreak. *Journal of Travel Medicine* (2020); 27(3):taaa031.

69 Ogbodo, JN, Onwe, EC, Chukwu, J, Nwasum, CJ, Nwakpu, ES, Nwankwo, SU, et al. Communicating health crisis: A content analysis of global media framing of COVID-19. *Health Promotion Perspectives* (2020);10(3):257–269.

70 McNaughton-cassill, ME. The news media and psychological distress. *Anxiety, Stress and Coping*, (2001);14:2, 193–211.

71 Lappe, M, Epigenetics, media coverage, and parent responsibilities in the post-genomic era. *Current Genetic Medicine Reports* (2016);4(3):92–97.

72 See for instance, Campbell, M. An increasing risk of family violence during the Covid-19 pandemic: Strengthening community collaborations to save lives. *Forensic Science International: Reports* (2020);2:1–3; Van Gelder, N, Peterman, A, Potts, A, O’Donnell, M, Thompson, K, Shah, N, et al. COVID-19: Reducing the risk of infection might increase the risk of intimate partner violence. *EClinical Medicine* (2020); 21:1–2.

73 Usher, K, Jones, CB, Bullar, N, Durkin J, Gyamfi, N, Fatema SR, et al. COVID-19 and family violence: Is this a perfect storm? *International Journal of Mental Health Nursing* (2021);30(4):1–11.

74 Gildner, T and Thayer, Z. Maternal and child health during the COVID-19 pandemic: Contributions in the field of human biology. *American Journal of Human Biology* (2020); 32:e23494.

75 Paremoer, L, Nandi, S, Serag, H and Baum F. Covid-19 pandemic and the social determinants of health. *BMJ* (2021);372:n129.

76 McElroy, E, Patalay, P, Moltrecht, B, Shevlin, M, Shum, A, Creswell, C and Waite, P. Demographic and health factors associated with pandemic anxiety in the context of COVID-19. *British Journal of Health Psychology* (2020);25(4):934–944.

77 See note 62, Sahin and Kabakci (2020, p.166).

78 See above.

79 Harrison, A, Taylor, N, Shields, N and Frawley H. Attitudes, barriers and enablers to physical activity in pregnant women: a systematic review. *Journal of Physiotherapy* (2018);64(1):24–32.

80 University of Virgina Health System, Exercise during pregnancy may save kids from health problems as adults. *Science News* (2021) March 15. Available at: https://www.sciencedaily.com/releases/2021/03/210315160706.htm (last accessed 17 July 2021).

81 Cheu, L, Yee, L and Kominiarek, M. Food insecurity during pregnancy and gestational weight gain. *American Journal of Obstetrics & Gynecology* (2020);2(1):100,068.

82 Dolin, C, Compher, C, Oh, J and Durnwald C. Pregnant and hungry: addressing food insecurity in pregnant women during the COVID-19 pandemic in the United States. *American Journal of Obstetrics & Gynecology* (2021);3(4):100,378.

83 See above.

84 Barbosa-Leiker, C, Smith, CL, Crespi, EJ, Brooks, O, Burduli, E, Ranjo S, et al. Stressors, coping, and resources needed during the COVID-19 pandemic in a sample of perinatal women. *BMC Pregnancy and Childbirth* (2021);21:171.

85 Rollston, R and Galea, S. The coronavirus does discriminate: How social conditions are shaping the COVID-19 pandemic. *Harvard Medical School* (2020) May 5. Available at http://info.primarycare.hms.harvard.edu/blog/social-conditions-shape-covid (last accessed 17 July 2021).

86 See note 54, Vears and D’Abramo (2018).
